# The role of apoptosis in cisplatin-induced ototoxicity in rats

**DOI:** 10.1016/S1808-8694(15)30528-0

**Published:** 2015-10-18

**Authors:** Marcos Rabelo De Freitas, Aline Almeida Figueiredo, Gerly Anne de Castro Brito, Renata Ferreira de Carvalho Leitao, Jose Valdir de Carvalho, Raimundo Martins Gomes, Ronaldo de Albuquerque Ribeiro

**Affiliations:** 1 Adjunct Professor of Otorhinolaryngology - Medical School of the Federal University of Ceará; 2 Substitute Professor of Otorhinolaryngology - Medical School of the Federal University of Ceará; 3 Associate Professor of Histology - Morphology Department- Medical School of the Federal University of Ceará; 4 Researcher - Department of Physiology and Pharmacology - Medical School of the Federal University of Ceará; 5 Medical Student - Federal University of Ceará; 6 Medical Student - Federal University of Ceará; 7 Associate Professor - Department of Physiology and Pharmacology - Medical School of the Federal University of Ceará. Head of the Cancer and inflammation physiology Lab, LAFICA

**Keywords:** apoptosis, hearing, cisplatin, hearing loss

## Abstract

Cisplatin is a chemotherapy agent frequently used to treat different types of neoplasia. Ototoxicity is one of the side-effects which cause significant morbidity and limits its use. This study aimed at assessing the role of apoptosis in cisplatin-induced ototoxicity.

**Design:**

experimental study.

**Materials and Methods:**

male Wistar rats were treated with intraperitoneal cisplatin, in the doses of 24 and 16 mg/kg. The animals were assessed by means of distortion product evoked otoacoustic emissions (DPEOAE) or brainstem evoked auditory potentials (BEAP) in the third (D3) and fourth (D4) days after drug infusion onset. Following that, their cochleas were removed for immunohistochemical studies of apoptosis - TUNEL method.

**Results:**

the group treated with 24 mg/kg showed a significant reduction in DPEOAE amplitude, and such fact was not seen with the 16 mg/kg. Both doses caused an increase in BEAP electrophysiological threshold in D3 and D4. Apoptosis was the injury mechanism responsible for the cisplatin-induced ototoxicity −16 mg/kg dose, when the animals were assessed on D3.

**Conclusion:**

apoptosis may be involved in the cisplatin-induced ototoxicity, depending on the dose and time of injury assessment.

## INTRODUCTION

The cochlea is the fundamental sensorineural structure in the peripheral auditory process of the acoustic message. It is made up by a series of complex epithelial structures, with sensorial and support cells located on the basilar membrane. Its damage, almost always irreversible, may have dire consequences for the affected individual, which may have his communication capacity compromised, with severe social impairment. On the other hand, despite the ototoxic potential of some antineoplastic drugs, cisplatin among them, they must not be disregarded as a treatment alternative for patients with malignant neoplasia. Thus the importance of establishing exactly the mechanisms through which the ototoxic effects of such drugs, in order to search for strategies to reduce such toxicity, without compromising its therapeutic effect^1^.

Cisplatin (cisdiaminodicloroplatinum - CCDP) is a frequently used chemotherapeutic agent in the treatment of many types of neoplasias, especially of the head and neck^2^. The antineoplastic action mechanism is associated to the selective and persistent inhibition of the deoxyribonucleic acid synthesis (DNA)^3^. Its side effects include ototoxicity, kidney toxicity, medullar suppression and gastrointestinal disorders^4^. These types of toxicity can impact treatment - as it reduces the chemotherapy dosage, frequency and duration for many patients^5^.

Cisplatin-induced ototoxicity was first described by Rossof et al., in 1972, and it has been broadly studied since then^6^. Its incidence seems to vary between 4 and 50%^7^, ^8^, ^9^, although alterations in the high frequency audiometry may be seen in almost all the cases^10^.

This type of lesion seems to result from free-radicals-induced damage to many tissues^11^, ^12^, ^13^, ^14^. It has been shown that oxygen reactive species are generated in the cochlea after exposure to cisplatin^15^ and that such oxidative stress can cause cochlear cell death by apoptosis secondary to the activation of caspase-3^16^.

Apoptosis is an active form of cell death which occurs during normal development, as well as when cells are exposed to certain types of attacking agents such as ischemia/hypoxia, radiation or toxin^17^ and this lesion mechanism seems to be the pathophysiological basis of cisplatin-induced ototoxicity.

Inner ear cell apoptosis can be triggered by the formation of complexes between cisplatin and the DNA of the damaged cell, preventing the progression of the cell cycle^18^, ^19^, ^20^. Another pathway proposed for cisplatin-induced ototoxicity in animal models establishes that the drug-induced oxidative stress would trigger a cascade of intracellular reactions which ultimately lead to apoptosis^13^.

Liu et al.^21^ and Cheng et al.^22^, through experiments with cultures of organ of Corti cells from 3 day-old Wistar rats showed the role of apoptosis as mechanism of cell lesion by cisplatin. Watanabe et al.^23^ identified fragmented DNA in the spiral ganglion and stria vascularis of guinea pigs submitted to treatment with 10 mg/kg of cisplatin. However, this finding was not seen in the organ of Corti hair cells. In the study by Alam et al.^24^ with Mongolian rodents, the identification of apoptotic cells after the administration of CCDP 20 mg/kg, happened in all cochlear structures, including the inner and outer hair cells, support cells, spiral ganglion cells, stria vascularis and spiral ligament. Devarajan et al.^18^ also showed evidence in culture of organ of Corti cells from rats, that CDDP promotes apoptosis in lower concentrations and that higher doses can lead directly to cell necrosis, and these two mechanisms can represent a continuum. Wang et al.^25^ showed that the 10 mg/kg dose of cisplatin was capable of inducing apoptosis to the cochleas of guinea pigs, especially in the outer hair cells, the inner hair cells and stria vascularis. They also reported that through transmission electron microscopy they noticed other lesion mechanisms such as autolysis and necrosis can be involved in cisplatin-induced ototoxicity.

The apoptosis of cochlear hair cells is dependent on the expression of pro-apoptotic proteins such as p5312, caspases^21^, ^22^, ^25^, calpain^26^, Bax and Bid^18^, c-jun NH2-terminal kinase (JNK)^22^ and antiapoptotic such as BCL-2^18^, ^24^. Therefore, to reduce inner ear cell apoptosis without inhibiting the antitumoral effect of CDCP can be a valid strategy to reduce the ototoxic effects of this chemotherapeutic agent^27^. This study aimed at assessing the role of apoptosis in the cisplatin-induced cochlear lesion in rats.

## MATERIALS AND METHODS

For our study we used male Wistar rats with weights varying between 200 and 348g, kept in cages with free access to food and water, in natural sleep and wake cycles, and handled according to the standards advocated by the Brazilian College of Experimentation with Animals (COBEA), found at the website: www.cobea.org.br. The project was submitted to the approval of the Ethics in Research with Animals Committee (CEPA), and was approved under protocol # 28/05.

The animals were divided in 8 groups according to dose, means of cisplatin administration, type and time of functional assessment (n= number of rats).

Group 1 (n=11): Rats treated with cisplatin at the dose of 8 mg/kg/day during 3 consecutive days (total of 24 mg/kg) and assessed before treatment (D0) and three days (D3) after its onset by distortion product evoked otoacoustic emissions (DPOAE).

Group 2 (n=6): Rats treated with saline solution at the dose of 8 ml/kg/day during 3 consecutive days (total of 24 ml/kg) and assessed before treatment (D0) and three days (D3) after its onset by distortion product otoacoustic emissions (DPOAE).

Group 3 (n=8): Rats treated with cisplatin at the dose of 8 mg/kg/day during 3 consecutive days (total of 24 mg/kg) and assessed before treatment (D0) and four days (D4) after its onset by distortion product otoacoustic emissions (DPOAE).

Group 4 (n=6): Rats treated with saline solution at the dose of 8 ml/kg/day during 3 consecutive days (total of 24 ml/kg) and assessed before treatment (D0) and on the 4th day after its onset (D4) by distortion product otoacoustic emissions (DPOAE).

Group 5 (n=12): Rats treated with cisplatin in a single dose of 16 mg/kg/day and assessed before treatment (D0) and three days (D3) after its onset by distortion product otoacoustic emissions (DPOAE).

Group 6 (n=5): Rats treated with saline solution in a single dose of 16 ml/kg/day and assessed before treatment (D0) and three days (D3) after its onset by means of distortion product otoacoustic emissions (DPOAE).

Group 7 (n=7): Rats treated with cisplatin in a single dose of 16 mg/kg/day and assessed before treatment (D0) and four days (D4) after its onset by means of distortion product otoacoustic emissions (DPOAE).

Group 8 (n=6): Rats treated with saline solution in a single dose of 16 ml/kg/day and assessed before treatment (D0) and four days (D4) after its onset by means of distortion product otoacoustic emissions (DPOAE).

In a parallel study, similar animals were divided into four groups in an attempt to assess its pre and post treatment thresholds and hearing by means of brainstem evoked auditory potentials (BEAP), thus being:

Group 9 (n=11): Rats treated with cisplatin in the dose of 8 mg/kg/day for 3 consecutive days (total of 24 mg/kg) and evaluated before treatment (D0), three (D3) and four days (D4) after its onset by brainstem evoked auditory potential (BEAP).

Group 10 (n=7): Rats treated with saline solution in the dose of 8 ml/kg/day for 3 consecutive days (total of 24 ml/kg) and assessed before treatment (D0), three (D3) and four days (D4) after its onset by brainstem evoked auditory potentials (BEAP).

Group 11 (n=12): Rats treated with cisplatin in a single dose of 16 mg/kg/day and assesses before treatment (D0), three (D3) and four days (D4) after its onset by means of brainstem evoked auditory potentials (BEAP).

Group 12 (n=8): Rats treated with saline solution in a single dose of 16 ml/kg/day and assessed before treatment (D0), three (D3) and four days (D4) after its onset by brainstem evoked auditory potentials (BEAP).

The Wistar rats were submitted to deep anesthesia by ketamine 50 mg/kg associated with xylazine 10 mg/kg. A prior otoscopy was carried out eliminating the animals with signs of middle and outer ear disorders. Those with normal otoscopy were submitted to distortion product evoked otoacoustic emissions (DPEOAE), immediately before the administration of the drugs. In the 8 mg/kg cisplatin for 3 consecutive days or 8 ml/kg saline solution for 3 consecutive day's groups, the drugs were injected in the peritoneum immediately after the conclusion of the first auditory assessment. In the two subsequent days, after a new weighing of the rats, 8 mg/kg cisplatin or 8 ml/kg saline solution were administered again, in order to result in a final respective dose of 24 mg/kg and 24 ml/kg. Twenty four (D3) or 48h (D4) after the last administration, the rats were anesthetized again, a new otoscopy was carried out in order to rule out those with external or middle ear disorders during the period of drug administration, and were submitted to a new auditory evaluation by DPEOAE. In the groups in which cisplatin was injected by intraperitoneal via in the single dose of 16 mg/kg and 16 ml/kg saline, we used a Kd Scientific series 100 infusion pump in order to fix the infusion time to 30min. When necessary, a new dose of anesthetics was injected. Auditory evaluation was also carried out on the third (D3) or fourth (D4) days after drug administration.

Immediately after the last auditory evaluation by DPEOAE, we removed the right temporal bone, after slaughtering, by guillotine beheading. The cochlea was dissected by the immunohistochemical technique as described in subsequent items.

In the four last groups (9 to 12), the animals were submitted to the same procedures of anesthesia, otoscopy and injection of drugs aforementioned, without, however, removing temporal bones for immunohistochemical purposes. The auditory assessment was carried out by means of BEAP (Brainstem Evoked Auditory Potential), and the same group of animals was submitted to the exam immediately before and on the third and fourth days after the administration of the drugs.

The distortion product evoked otoacoustic emission tests (DPEOAE) were carried out by means of a MADSEN Capella - GN Otometrics otoacoustic emissions device, in a silent environment. The rats were anesthetized and in their right external auditory meatus we inserted the device's probe, using probes which are used with newborn babies. The stimulus encompassed 2 pure tones (F1 and F2) which F1/F2 frequency ratio was equal to 1.22. The stimulus intensity was fixed to 70 dB SPL. We analyzed a total of 1000 inputs. The resulting otoacoustic emissions were assessed in the frequencies of 3, 4, 6 and 8 kHz. We considered the presence of DPEOAE for a signal/noise (S/N) ratio of, at least 6 dB SPL, according to the technical specifications of the device used.

In order to perform the brainstem evoked auditory potential test (BEAP) we used the Interacoustic EP 25 device in a silent room. With the animals anesthetized, platinum subdermal electrodes were placed on the vertex (positive), right retroauricular region (negative) and nasal tip (ground). ER-3A insertion earphones were coupled to the probe used to do auditory assessment of newborns and were introduced in the right external auditory meatus of the rats. The stimuli employed were rarefaction clicks, sounded at a rate of 15 per second, with a total of 700 clicks during a 15msec analysis time. The passing band used was from 0 to 3,000 Hz. The stimuli started at 80 dB HL and were progressively reduced until complete wave disappearance. For the electrophysiological auditory threshold, we considered the lowest stimulus intensity in which we could notice the wave II.

In the immunohistochemistry for apoptosis by the TUNEL (TdT-mediated dUTP nick end-labeling) method, four 4mm slices of the cochlea were made, along the modiulus, and were prepared in slides covered with L-polylysine. For this technique we used the ApopTagR S 7100 (Chemicon International) kit. The histological cross-sections had the paraffin removed by xylol and were rehydrated with decreasing concentrations of alcohol (absolute, 95% and 70%). Following that, they were flushed in phosphate buffer solution (PBS) and incubated at room temperature with proteinase K for 15min in order to promote antigenic recovery. After flushed with distilled water, the material was treated with 3% hydrogen peroxide volume per volume in PBS during 5min, in order to block endogenous peroxidase. A new flushing with PBS was carried out and afterwards the histology cross-sections were incubated with Equilibration Buffer for 10s and immediately after with the TdT (desoxynucleotidil transferase) enzyme, associated with the nucleotides marked with digoxygenin (reaction buffer) which would bind to the free fragmented DNA hydroxyls from the apoptotic cells. The negative control was not treated with the enzyme. The material remained in the oven at 37°C for 1h and the reaction was concluded with the stop solution. Flushing with PBS, following that the addition of antidigoxygenin tandem solution and incubation at room temperature for 30 minutes. Another PBS flushing and added to DAB (diaminobenzidin), the peroxidase substrate, then incubated for 3 to 6min, in order to develop the reaction color. Flushing with distilled water, immersion in 100% N-butanol and xylol and, after that, the slides were setup with EntelanR.

**Figure 1:**
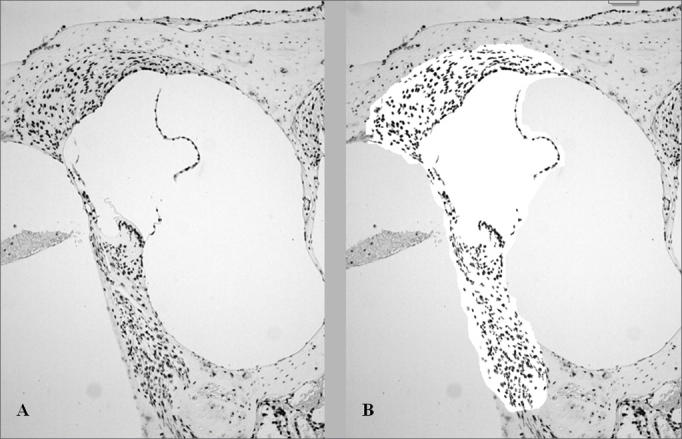
Histomorphometric analysis, after TUNEL immunohistochemistry technique, of an animal treated with 16mg/kg of cisplatin, which had its cochlea removed on D3. The brown color indicates cell death. A. Color seen before selection and quantification of the dyed area. B. After selection of the area to be analyzed by the SAMH 1.0 software, isolating the brown points.

In order to quantify the color intensity of the structures, we made microphotographies of the basal turn with a 100x magnification through the Leica DFC 320 digital capture system. Using the Histomorphometric Analysis System software (SAHM 1.0), an area corresponding to the middle ramp of the Corti spiral organ, spiral ganglion, stria vascularis and spiral ligament was selected and assessed the dyed area over non-dyed area ratio (pixels/pixels). The SAHM 1.0 software selects a pre-defined color one wishes to evaluate and quantifies the area occupied by such color (Fig. 1).

In order to make the graphs and do the statistical analysis we used the GraphPad Prism 4.00.255 software.

We assessed the normal sample distribution through the Komogorov-Smirnov test. Results were expressed as mean ± the mean standard error (MED ± EPM), for the continuous data, and as median (Md) and minimum (Min) and maximum (Max) values for the ordinal data. The minimum significance accepted was 5%. The experimental procedures were compared using the following tests:

T-Student test in order to compare the distortion product otoacoustic emission mean values in each frequency before and after treatment and to compare the dyed area fraction in relation to the non-dyed area by immunohistochemistry following the TUNEL method in each group treated with cisplatin and its control.

ANOVA variance analysis with significance between the groups established by the Tukey test in order to compare the mean values of the electrophysiological thresholds of the animals obtained by means of the brainstem evoked auditory potential on the first (D0), fourth (D3) and fifth (D4) days of assessment.

## RESULTS

Functional hearing assessment by Distortion Product Evoked Otoacoustic Emissions (DPEOAE)

**Figure 2:**
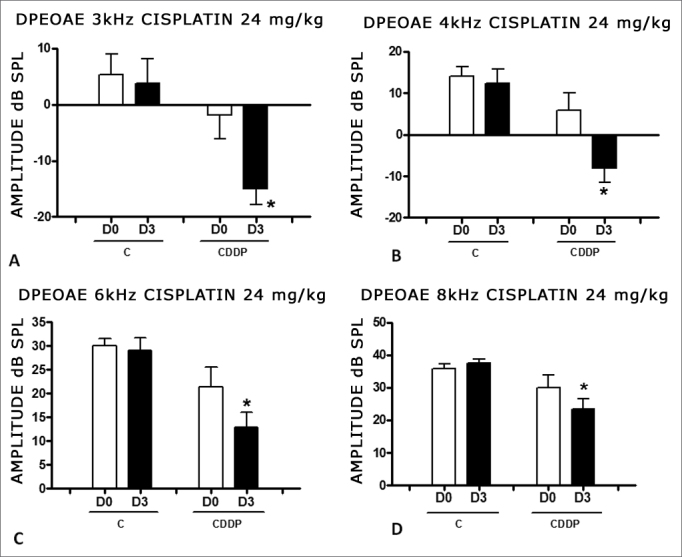
Graph showing the DPEAOAE amplitude values as mean ± mean standard error on days D0 and D3, for groups 1 and 2. The asterisk represents statistical significance. CDDP equals; cisplatin; C equals; control. Graph A: frequency of 3 kHz (T test T: * p equals; 0.0095 - D3 × D0 CDDP). Graph B: 4 kHz frequency (T -Test: * p equals; 0.0073 - D3 × D0 CDDP). Graph C: 6 kHz frequency (T Test: * p equals; 0.0284 - D3 × D0 CDDP). Graph D: 8 kHz frequency (T test: * p equals; 0.0338 - D3 × D0 CDDP).

We found a significant reduction in the DPEOAE amplitude within the frequencies tested (3, 4, 6 and 8 kHz) between D0 and D3 for the group that received cisplatin at the dose of 24 mg/kg (Group 1). Such reduction was not found in the control group. (Figs. 2 A, B, C and D).

**Figure 3:**
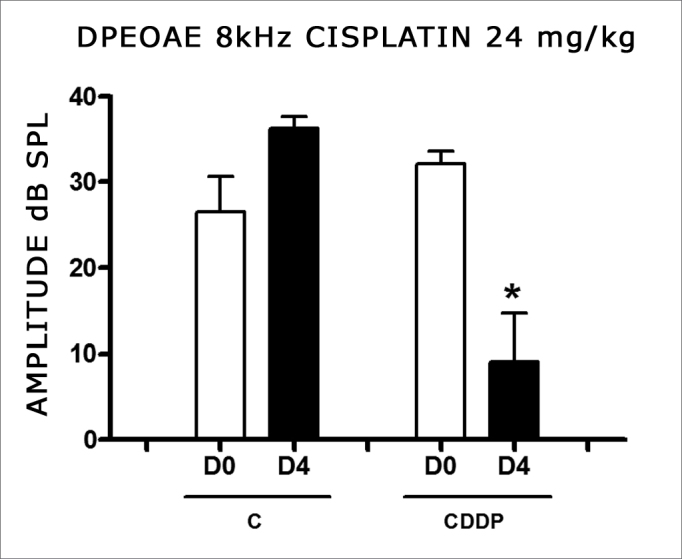
Graph of the DPEAOAE amplitude values expressed as mean ± mean standard error on days D0 and D4, of groups 3 and 4 in the 8 kHz frequency. The asterisk represents statistical significance. CDDP equals; cisplatin; C equals; control. T- Test: * p equals; 0.0459 (D4 × D0 CDDP).

Group 3 showed a high animal mortality on D4 after the procedure. Of the eight animals which received cisplatin in the cumulative dose of 24 mg/kg, only 3 remained alive for the DPEOAE test on D4. Of these, 2 had no DPEOAE, proven by a signal/noise ratio difference lower than 6 dB SPL, in the frequencies of 3 and 4 kHz. One animal also did not show DPEOAE in the 6 kHz frequency. In the 8 kHz frequency we noticed a significant reduction in DPEOAE amplitude between D0 and D4 (Fig. 3).

**Figure 4:**
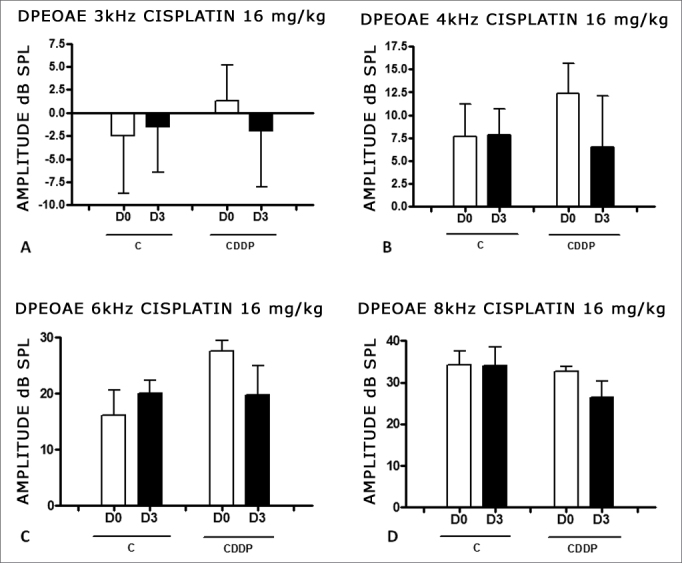
Graph of the DPEAOAE amplitude values expressed as mean ± mean standard error in the averages of days D0 and D3, for groups 5 and 6. CDDP equals; cisplatin; C equals; control. There was no statistical significance in any of the groups (T test: p > 0.05).

**Figure 5:**
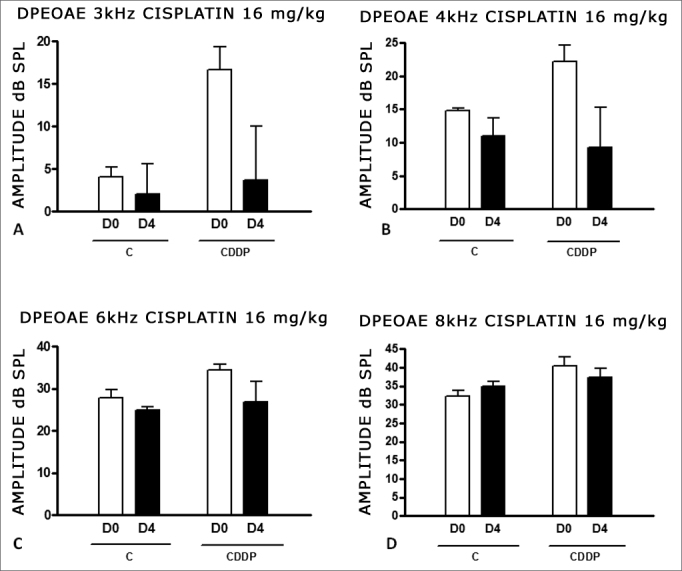
Graph of DPEAOAE amplitude values expressed as mean ± mean standard error in the averages of days D0 and D4, of groups 7 and 8. CDDP equals; cisplatin; C equals; control. There was no statistical significance in any of the groups (T test: p > 0.05).

With the dose of 16 mg/kg of cisplatin, it was not possible to identify a statistically significant reduction in DPEOAE mean amplitude values between D0 and D3 and between D0 and D4, in all the frequencies studied (p > 0.05) (Figs. 4 A, B, C and D and 5 A, B, C and D).

### Functional hearing assessment by brainstem evoked auditory potential (BEAP)

**Figure 6:**
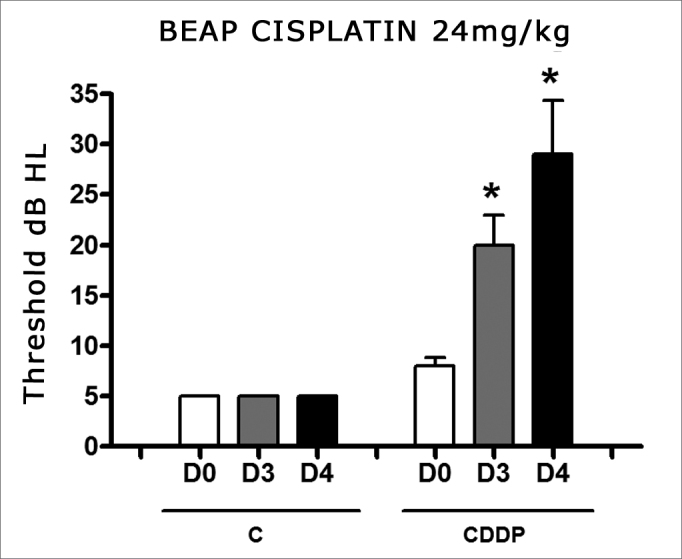
Graph showing the mean electrophysiologic thresholds of groups 9 and 10 in D0, D3 and D4, expressed as mean ± mean standard error (MED ± EPM). The asterisk represents a statistically significant difference. CDDP equals; cisplatin; C equals; control. ANOVA - Tukey: * p < 0.01 (D3 × D0 CDDP); * p < 0.001 (D4 × D0 CDDP).

There was a significant increase in the mean electrophysiological threshold in the animals injected with 24 mg/kg cisplatin on D3 and D4 when compared to D0. We did not notice a statistically significant difference of these thresholds between D3 and D4. In the control group there was no elevation of the electrophysiological thresholds (Fig. 6).

**Figure 7:**
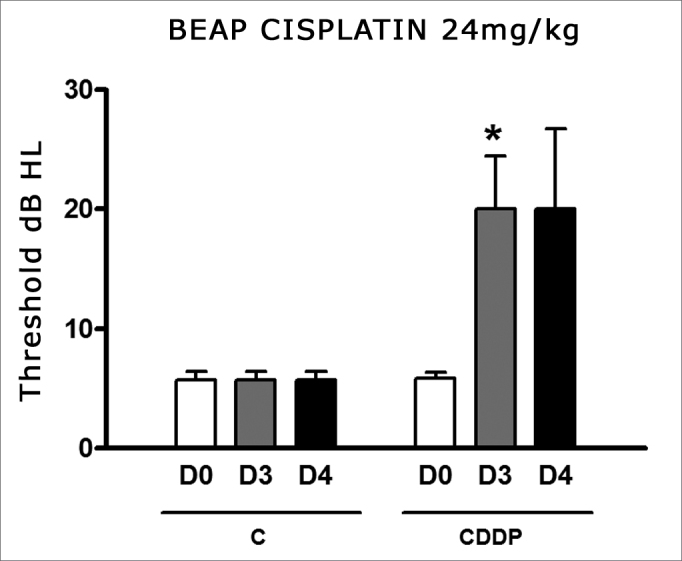
Graph showing the mean electrophysiologic thresholds of groups 11 and 12 in D0, D3 and D4, expressed as mean ± mean standard error (MED ± EPM). The asterisk represents a statistically significant difference. CDDP equals; cisplatin; C equals; control. ANOVA - Tukey: * p < 0.05 (D3 × D0 CDDP).

We noticed a significant increase in the mean electrophysiological threshold of the animals injected 16 mg/kg cisplatin on D3 when compared to D0. We did not notice statistically significant differences of these thresholds between D3 and D4. In the control group there was also no elevation in the electrophysiological thresholds (Fig. 7)

### Apoptosis in Cisplatin-Induced Ototoxicity

**Figure 8:**
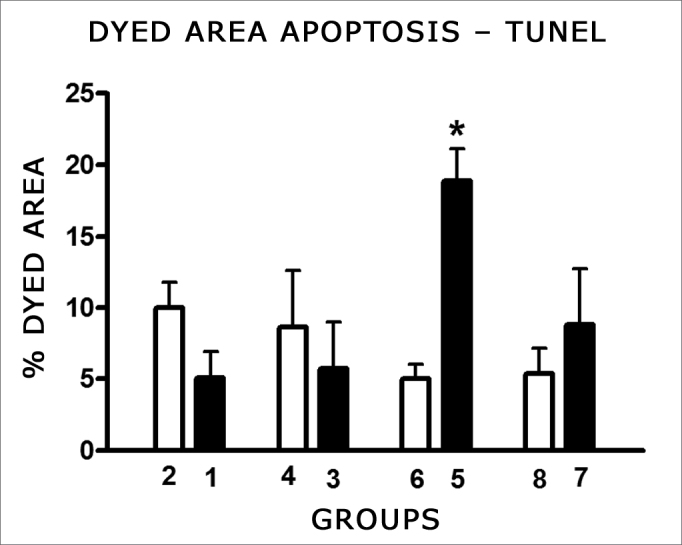
Graph showing the mean fraction ± mean standard error (MED ± EPM) of the colored area (TUNEL positive) in the cochlea basal turn of the animals from the different groups. The asterisk represents statistical significance. T-test: * p equals; 0.0013 (Group 5 - CDDP 16 D3 DPEAOAE × Group 6 - control).

Cells were marked by the TUNEL method in the areas of the membranous labyrinth (inner and outer hair cells, stria vascularis, spiral ligament, spiral limbos, spiral ganglion) for both doses and in the control groups. Comparing the color intensity between each treated group and its control with the SAHM 1.0 software we noticed a significantly larger number of marked cells by the TUNEL method on the cochleas of the animals treated with 16 mg/kg cisplatin, which had their temporal bones removed on D3. There was no significant difference between the other groups and their controls (Fig. 8).

## DISCUSSION

Cisplatin is one of the most broadly used and effective chemotherapeutic agents against epithelial malignant tumors, including testicle, ovary, gallbladder, lung head and neck^19^. Some of its side effects, such as nausea, vomits, renal dysfunction, can be controlled with the use of serotonin receptor antagonists and hydration^28^. Nevertheless, ototoxicity remains as one of the side effects which cause significant morbidity and which frequent curbs its use^29^. For this reason, we need to develop experimental models in order to better understand the mechanisms involved in cisplatin-induced toxicity and many feasible ways to minimize it.

Functional hearing evaluation of rats in the experiment was first carried out through the DPEOAE - distortion product evoked otoacoustic emissions, since it is the only method available to date with which the study could be carried out. During the experiments, we acquired a BEAP device, which made possible to carry out a functional assessment of hearing in rats by brainstem evoked auditory potential. Thus, it was not possible to submit the same group of animals to both methods of functional evaluation.

In the present study, it was only the 24 mg/kg dose that was capable of triggering a cisplatin-induced ototoxicity measurable by DPEOAE in all the frequencies studied.

When the functional assessment was carried out through the brainstem evoked auditory potential (BEAP), we noticed an increase in the electrophysiological threshold in the two doses employed.

Some studies suggest that cisplatin can cause cell damage because it interacts with DNA chain nucleophilic sites, triggering mechanisms which result in apoptosis19. This effect, which is beneficial in terms of the neoplasia treatment, is the very basis for understanding the ototoxicity of this drug^20^.

Another mechanism proposed to explain this cisplatin-induced adverse effect is through the oxidative stress which would cause irreversible cell damage and apoptosis^11^, ^13^, ^17^, ^18^, ^25^, ^26^, ^30^.

In the present study we were able to use the immunohistochemistry TUNEL method (ApopTagR) to detect DNA fragmented chains and establish that the cochleas of rats treated with 16 mg/kg of cisplatin and which had their temporal bones removed 3 days later were significantly marked when compared to the controls. All the structures of the membranous labyrinth were marked, including the inner and outer hair cells, support cells; stria vascularis, spiral ganglion, spiral ligament, spiral limbos, and these results were similar to those reported by Alam et al.24 in Mongolian rodents. Despite the 24 mg/kg dose and the animals assessed on D3 and D4, as well as the dose of 16 mg/kg assessed on D4, the cells marked by TUNEL were also in evidence, their color intensity was not different from the respective controls. These findings lead us to believe that the lower dose, for a shorter assessment time was capable of triggering cell apoptosis, while higher doses or a longer evaluation time were associated with other mechanisms of cell death such as autolysis or necrosis. Studies carried out by Liu et al.21 and Cheng et al.^31^ in cultures of P-3 Wistar P-3 rat cells also showed a TUNEL arking pattern with the use of cisplatin. Devarajan et al.^18^, used the TUNEL method (ApopTagR) also in cultures of organ of Corti cells, proposed that lower cisplatin doses led to cell death by apoptosis, while larger doses led straight to necrosis, and these two mechanisms of cell death are a continuum. A study with guinea pigs in inner ear surface preparation showed the DNA fragmentation TUNEL pattern in outer hair cells and stria vascularis and less DNA fragmentation in inner hair cells with the use of 3 mg/kg cisplatin during five consecutive days^32^. These authors have also identified other mechanisms of cell damage, other than apoptosis, through the use of transmission electron microscopy. Some cells had vacuolated cytoplasm and intact cytoplasmatic membrane, suggesting autolysis. Others showed necrosis signs such as cell debris and cytoplasmic membrane disintegration.

The cochlear cell color in the control group is probably related to the cell lesion triggered by the very method used to remove this organ through beheading of the animal.

With this, apoptosis is part of the cell damage mechanisms in cisplatin-induced ototoxicity, depending on the dose and time of assessment used. Moreover, cells in apoptosis, if its natural evolution is followed, could have necrosis as final outcome.

## CONCLUSION

The 16 mg/kg cisplatin dose with assessment on the third day after its administration had apoptosis as mechanism of cell damage.

For higher doses of cisplatin (24 mg/kg) or assessment on the fourth day after its administration, other lesion mechanisms, different from apoptosis, may be involved.
